# Biogeographical Patterns of Coral‐Associated Symbiodiniaceae Across Thermal Regimes Including Trace Molecular Detection of *Cladocopium thermophilum* (C3‐Gulf) in the Seychelles

**DOI:** 10.1002/ece3.73941

**Published:** 2026-07-26

**Authors:** Reem K. AlMealla, Brisneve Edullantes, Boyd McKew, Kirsty Matthews Nicholass, Leanne J. Hepburn, Bethan Greenwood, David J. Smith, Jamaluddin Jompa Adalah, Gilberte Gendron, Michelle L. Taylor

**Affiliations:** ^1^ School of Life Sciences University of Essex Colchester Essex UK; ^2^ Nuwat for Environmental Research & Education Al Janabiyah Kingdom of Bahrain; ^3^ Department of Biology and Environmental Science, College of Science University of Philippines Cebu Lahug Cebu Philippines; ^4^ School of Biological and Marine Sciences University of Plymouth Plymouth UK; ^5^ Department of Fisheries Hasanuddin University Makassar Sulawesi Indonesia; ^6^ Island Biodiversity and Conservation Centre University of Seychelles Victoria Seychelles; ^7^ Bee Ecological Consulting Victoria Seychelles

**Keywords:** Arabian Gulf, biogeography, coral, genomics, Gulf Cooperation Council, symbiont

## Abstract

Reef‐building corals are under serious threat from ocean warming, which directly impacts their symbiotic partnership with dinoflagellates from the family Symbiodiniaceae; a partnership integral for the survival of coral reefs. Symbiodiniaceae types vary in their levels of tolerance to heat and light, with some reported to enhance a coral's ability to withstand thermal stress. This study investigated the composition and diversity of Symbiodiniaceae across different thermal regimes and latitudinal gradients covering three bioregions: Bahrain (Arabian Gulf—AG), the Seychelles (Western Indian Ocean—WIO), and Indonesia (Central Indo‐Pacific—CIP). Coral fragments from 11 coral host species (*n* = 183 colonies collected; *n* = 157 retained following sequencing quality control) were sampled across six reef sites spanning these regions. Results acquired through next generation sequencing (NGS) targeting the ITS2 rDNA region identified three main genera: *Cladocopium* (dominant across all sites), *Durusdinium*, and *Symbiodinium.* Observed biogeographical patterns suggested lower Symbiodiniaceae diversity and richness in the higher latitude and more thermally extreme reefs of Bahrain compared to Indonesia and the Seychelles. Shifts in Symbiodiniaceae community composition from thermally sensitive ITS2 types in reefs under comparatively lower thermal stress (Degree Heating Weeks (DHW) ≤ 4°C‐weeks) toward more thermotolerant ITS2 types in reefs exposed to higher thermal stress (DHW > 4°C‐weeks) were observed. Our results provide the first ITS2‐based molecular characterization of Symbiodiniaceae communities in Bahrain and document the widespread occurrence of the thermotolerant C3‐Gulf (*Cladocopium thermophilum*) type across sampled Bahraini coral hosts. In addition, the C3‐Gulf ITS2 type was detected at trace relative abundance (0.1%) within a coral sample from the Seychelles extending previous observations beyond AG, although the ecological significance of this low‐abundance detection remains uncertain. Overall, the findings provide baseline insights into coral‐Symbiodiniaceae associations across contrasting reef systems and highlight how symbiont community composition may vary under differing environmental, thermal, and biogeographic conditions.

## Graphical abstract

Reef‐building corals rely on symbiotic dinoflagellates (Symbiodiniaceae) for survival; however, the distribution of thermotolerant symbionts across environmental gradients remains poorly understood. Using ITS2 metabarcoding across three bioregions spanning extreme to moderate thermal regimes (Bahrain, Seychelles, and Indonesia), we show that Symbiodiniaceae community composition is structured by host identity, thermal regime, and latitude. We provide the first molecular confirmation of the thermotolerant C3‐Gulf lineage (*Cladocopium thermophilum*) in Bahrain and its first documented detection in the Western Indian Ocean, extending its known biogeographical range.

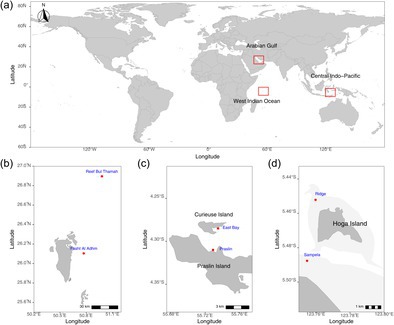

## 1 Introduction

Corals host a complex and diverse composition of microbes including microalgae, bacteria, viruses, fungi and archaea in their mucus layer, skeleton, and tissues (Blackall et al. 2015). This collective diverse and dynamic group of microbial communities, together with the coral host, is referred to as the coral holobiont (Rohwer et al. 2002; Blackall et al. 2015; Thompson et al. 2015). These microorganisms provide their host with benefits via various mechanisms, including photosynthesis, nitrogen fixation, the provision of nutrients and infection prevention, all of which contribute toward coral health (Trench 1979; Rohwer et al. 2002; Rosenberg et al. 2007). Among reef‐building (zooxanthellate) corals, these dinoflagellates of the family Symbiodiniaceae are particularly abundant, with their densities reaching several million per square centimeter of host tissue (LaJeunesse 2002). The coral holobiont functions as a dynamic system whereby external environmental conditions determine its members (Shashar et al. 1993; Tanner 1996; Thompson et al. 2015; Roik et al. 2016). Therefore, any change in environmental conditions will change the relative abundance of microbial species to facilitate the coral holobiont to adapt to the new condition (Reshef et al. 2006). Changes in environmental conditions alter the selective pressures on microbial species within the coral holobiont, leading to shifts in the community composition toward more resilient and better‐adapted microbes. This dynamic adjustment enhances the coral's ability to cope with and adapt to new environmental stresses (Meron et al. 2012). The mutualistic association between hermatypic corals (reef‐building) and their endosymbiotic Symbiodiniaceae (commonly known as zooxanthellae) is said to underpin the survival of coral reefs (Hume et al. 2015; Rouzé et al. 2017; LaJeunesse et al. 2018). Coral bleaching refers to the partial or complete loss of Symbiodiniaceae species from coral tissues, which occurs when corals are subjected to severe environmental stress that disrupts the coral‐algal symbiosis (Hoegh‐Guldberg 1999; Donner et al. 2005; Hoegh‐Guldberg et al. 2007). While elevated sea surface temperatures are considered the primary driver of mass coral bleaching events globally, other stressors including cold‐water anomalies, changes in salinity, irradiance, sedimentation and pollution may also induce bleaching (Paz‐García et al. 2012). Symbiodiniaceae inhabit vacuoles, the symbiosome, which are located within the endoderm cells of the coral polyp tissue (Trench 1979). Corals receive from the Symbiodiniaceae products of photosynthesis that are required for primary metabolism, such as sugars, fatty and amino acids, carbohydrates and small peptides, which contribute toward coral nutritional provision (Trench 1979; Papina et al. 2003; Morris et al. 2019). This energy supply from Symbiodiniaceae relates to the amount of energy available for coral calcification (Jones and Berkelmans 2010). In return, the Symbiodiniaceae receive crucial nutrients such as ammonia and phosphate, wastes from coral metabolism (Furla et al. 2000; Al‐Hammady 2013). An increase by 1°C or 2°C above the thermal tolerance threshold is often enough for corals to expel their associated Symbiodiniaceae (Lesser 2007; Desalvo et al. 2008; Ricaurte et al. 2016). This phenomenon can be reversed with no major consequences to the coral, depending on the length of exposure and severity of stress exposure (Baker et al. 2008).

The last 20 years of molecular technique advancements have revealed an array of Symbiodiniaceae diversity hosted within cnidarians and invertebrates (Rowan and Powers 1991; LaJeunesse 2001, 2002; Pochon et al. 2001; Coffroth and Santos 2005; Sampayo et al. 2008; Hill et al. 2011). Phylogenetic analyses based on the 18S rDNA and internal transcribed spacer (ITS) regions identified nine major Symbiodiniaceae lineages, historically referred to as clades A‐I, which are further subdivided into ITS2 types (Rowan and Powers 1991; LaJeunesse 2001, 2002; Pochon et al. 2006; Pochon and Gates 2010; Hill et al. 2011; Yang et al. 2012; Rouzé et al. 2017). Recent taxonomic revisions formally reclassified these clades (hereafter referred to as types) into distinct genera and species based on their genetic, physiological, and ecological variation (LaJeunesse et al. 2012, 2018). Over the years, numerous species of Symbiodiniaceae have been formally described, classified, and named based on ongoing taxonomic revision and molecular characterization efforts (Table S1; Trench and Blank 1987; Jeong et al. 2014; Lajeunesse et al. 2014, 2015, 2018; Hume et al. 2015; Parkinson et al. 2015; Ramsby et al. 2017).

Symbiodiniaceae types vary in their levels of tolerance to heat and light (Stat et al. 2006; Hennige et al. 2011; Silverstein et al. 2012). Coral‐Symbiodiniaceae species associations have been seen to include mono or multi‐clade associations (Fabina et al. 2012; Silverstein et al. 2012; Rouzé et al. 2017), with their type and ecological dominance influenced by regional and local environmental factors (Baker 2003; Ziegler et al. 2017). Corals are seen to be most commonly associated with Symbiodiniaceae type A‐D (LaJeunesse 2001), rarely with F and G (Ramsby et al. 2017).

Bleaching has been proposed to be a survival mechanism used by corals to overcome severe stress through altering the composition of their associated Symbiodiniaceae, a concept termed the “Adaptive Bleaching Hypothesis” (Buddemeier and Fautin 1993). This can occur through two primary mechanisms: either switching, in which the coral host acquires new, more thermally tolerant symbiont types from the surrounding environment; or shuffling, where thermally sensitive symbionts are expelled and previously rare, more stress‐tolerant types already present within the coral tissue increase in relative abundance (Rowan and Powers 1991; Baker et al. 2004; Palumbi et al. 2014; Rouzé et al. 2017; Guibert 2024). Shuffling relies on existing symbiont diversity within the host, while switching involves external uptake. Some studies suggest that shuffling incurs little to no physiological cost to the host (Abbott et al. 2021). For example, in a study on a common Indo‐Pacific branching coral species, *Acropora millepora* , it was found that switching to thermally tolerant Symbiodiniaceae species type D increased the coral's thermal tolerance between 1.0**°**C and 1.5**°**C (Berkelmans and Van Oppen 2006). However, the capacity to transition to thermally tolerant symbionts comes with a cost associated with physiological trade‐offs. Prior research suggests that reef‐building corals hosting stress‐tolerant Symbiodiniaceae types may exhibit reduced growth or calcification rates, a likely consequence of the energetic costs involved in maintaining these partnerships, especially under thermal stress or post‐bleaching conditions (Little et al. 2004; Mieog et al. 2009; Jones and Berkelmans 2010; Pettay et al. 2015). Notably, within the Symbiodiniacae, the genus *Durusdinium* (formerly clade D) is frequently associated with thermally extreme environments and is often cited in such trade‐offs. Hosts associating with *Durusdinium* tend to survive higher temperatures but typically exhibit slower growth rates compared to those hosting more thermally sensitive genera like *Cladocopium* (Jones and Berkelmans 2010; Chan et al. 2023). Nevertheless, exceptions exist, some *Durusdinium* lineages appear to confer thermal tolerance without significantly compromising host growth, particularly when these symbioses have co‐evolved over time (Chan et al. 2023). This highlights the complexity and specificity of host‐symbiont interactions and highlights the critical role that Symbiodiniaceae composition plays in coral resilience under ocean warming and acidification scenarios.

Subsequently, it is important to understand the diverse interactions among Symbiodiniaceae types and their hosts across varying environmental gradients, as these relationships may influence reef resilience and future reef accretion rates under climate change and sea level rise scenarios. This study examines the composition and diversity of Symbiodiniaceae species across different thermal regimes and latitudinal gradients in three bioregions, including the rarely sampled reefs of Bahrain. Based on previous studies, we expected thermally extreme environments to be associated with a greater relative abundance of thermotolerant Symbiodiniaceae types.

## 2 Materials and Methods

### 2.1 Site Selection and Description

**FIGURE 1:**
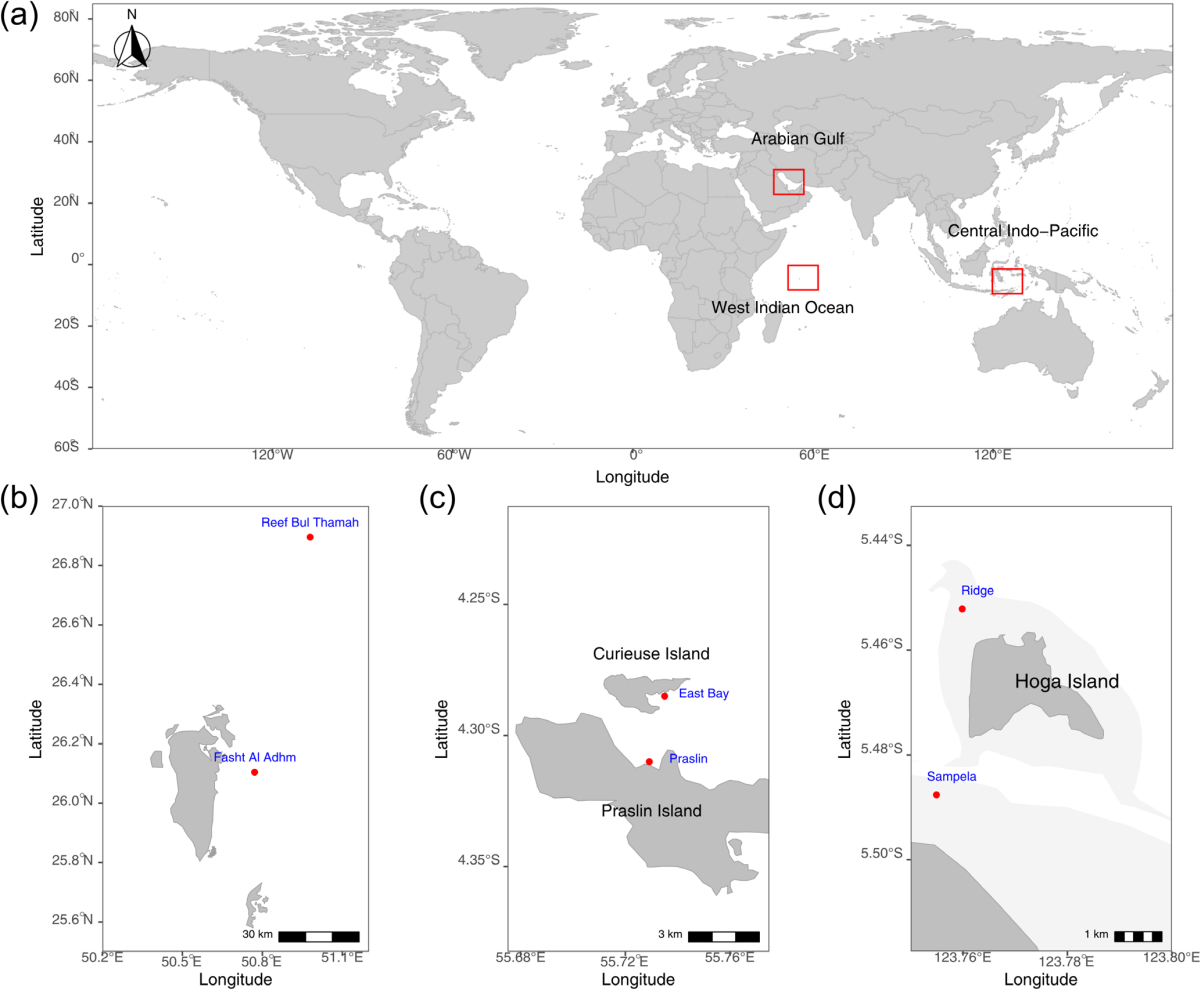
Location of Study Sites – (a) Location of Bahrain (Arabian Gulf), the Seychelles (Western Indian Ocean) and Indonesia (Central Indo‐Pacific); (b) Location of study sites within Bahrain; (c) Curieuse Marine National Park, Seychelles; (d) Hoga Island, Wakatobi Marine National Park, Indonesia.

This research focuses on six study sites located in three bioregions (two in each region): Bahrain which is located in the Arabian Gulf (AG), the Seychelles (Western Indian Ocean (WIO)) and Indonesia (Central Indo‐Pacific (CIP)) spanning a latitudinal gradient from approximately 26° N to 6° S (Figure 1). The sites have been characterized into three thermal regions based on data derived from the CoRTAD version 6 database^1^ (Table 1). These SST‐derived thermal metrics were used to characterize long‐term regional thermal regimes rather than represent exact environmental conditions at the time of biological sampling.

**TABLE 1:** Characterization of thermal regime in each of the bioregions (equations used to derive the presented thermal metrics were sourced from AlMealla et al. 2024; brief definitions of the variables^a^).

Thermal regime^a^	Study site	Min. SST	Max. SST	Annual mean	Min. climatology	Max. climatology	Seasonal range	Bleaching threshold	Max DHW	Mean DHW
High	Reef Bul Thamah (Bahrain)	16.5	36.2	26.2	19.1	33.1	14	34.1	18.2	4.8
	Fasht Al Adhm (Bahrain)	15.5	37	26	17.9	33.5	15.6	34.5	20.8	4.7
Moderate	Ridge (Indonesia)	24.5	32.7	28.5	26.5	29.7	3.2	30.7	6.1	2.2
	Sampela (Indonesia)	24.6	33.5	28.4	26.6	29.9	3.3	30.9	14.7	3.7
Low	East Bay (Seychelles)	23.4	32.2	28	25.8	30.1	4.2	31.1	6.5	1.7
	Praslin (Seychelles)	23.3	31.9	28	25.7	30	4.3	31.1	5.6	1.6

*Note:* All values represent temperature in °C with the exception of DHW which is in °C‐weeks.

^a^ *Thermal regimes* have been assigned based on the combination of the maximum SST, bleaching threshold, and DHW expressed on the reefs. *Climatology* the long‐term mean of SST conditions over time. *Seasonal Range* the difference between the minimum and maximum SST over time. *Bleaching threshold* is the value where SST have exceeded maximum long‐term mean by 1°C. *Degree Heating Week* (*DHW*) is defined as of accumulated daily hotspots over 12 consecutive weeks when the thermal stress anomaly is ≥ 1°C. DHW values of ≥ 4°C and < 8°C‐weeks, corresponds to delineation between coral bleaching and mortality levels (unit = °C‐weeks).

Reefs from both turbid and clear‐water (herein referred to as “optimal”) sites were chosen to represent common environmental gradients (Table 2). Reefs in Bahrain endure extreme sea surface temperatures (SST) ranging between 16**°**C and 35°C while reefs in the Seychelles and Indonesia experience fairly narrow SST differences ranging between 24**°**C and 29°C (Hume et al. 2013; Rowley et al. 2015), with El Niño events occurring irregularly at intervals of 3.5–5.5 years (Charles et al. 1997). This difference in thermal regime is said to influence the variations in diversity and richness of coral species in Bahrain and Indonesia (Coles 2003).

**TABLE 2:** Environmental characteristics of study sites (Bahrain: AlMealla et al. 2024).

Site	Site code	Depth (m)	Temp. (°C)	Salinity (ppt)	Sedimentation rates (g cm^−2^ day^−1^)^b^	Light attenuation (*K*_d_ PAR)^c^	Distance	Impacts
Bahrain (AG)								
Fasht Al Adhm (Turbid)	BH‐TB	5–7	16–35	42–44	0.23 ± 0.04	0.02	~11 km east of the main island	Heavily impacted due to various anthropogenic activities mainly reclamation and dredging
Reef Bul Thamah (Optimal)^a^	BH‐OP	10–12			0.19 ± 0.04	0.04	~80 km northeast of the main island	Although located within a Marine Protected National Park (MPA), the site is subjected to illegal fishing and destructive fishing practices
Seychelles (WIO) – Curieuse Marine National Park								
Praslin (Turbid)	SY‐TB	5–10	25–29	~35	0.03 ± 0.01	0.2–0.4	~1.5 km southwest of Curieuse Island	Site is subjected to large sediment load since it is located closer to Praslin island, which has intensive tourist marine activities
East Bay (Optimal)^a^	SY‐OP	5–12			0.04 ± 0.01	0.1–0.2	~1.5 km southeast of Curieuse Island	One of the least impacted sites with minimum anthropogenic impacts. Carbonate fringing reefs with minimal tourist activity
Indonesia (CIP) – Wakatobi Marine National Park, South East Sulawesi								
Sampela (Turbid)	IN‐TB	3–10	26–30	32–34	6.3 ± 2.7	0.01	~1.5 southwest Hoga Island	Site is adjacent to the Bajo village of Sama Bahari and is subjected to large sediment load and various anthropogenic activities, thus heavily impacted
Ridge (Optimal)^a^	IN‐OP	5–20			2.6 ± 1.0	0.02	~1 km northwest Hoga Island	One of the least impacted sites within the area with some artisanal line fishing occurring

^a^ Optimal in this study is referred to as clear waters.

^b^ *Bahrain*: Due to time constraints, sedimentation rates (g cm^−2^ day^−1^) were measured at a depth of 10 m using sediment traps (*n* = 6). Traps were deployed for a period of 3 days at each study site in May 2018 (AlMealla et al. 2024); *Seychelles*: Sedimentation rates (g cm^−2^ day^−1^) were measured at 10 m depth using sediment traps; however, due to expedition time constraints the traps were only deployed for a total of 4 days in April 2018; *Indonesia*: Sedimentation rates (mg cm^−2^ day^−1^) were collected by Operation Wallacea between 2006 and 2011 (mean values are reported here along with ±SD and taken from Franco 2014).

^c^ *Bahrain*: Sourced from AlMealla et al. (2024). *Seychelles and Indonesia*: Light intensity was measured using HOBO loggers deployed at two depths (5 and 10 m), and calibrated to calculate photosynthetically active radiation (PAR) following the methods outlined in Long et al. (2012). Light attenuation coefficients (*K*_*d*_(PAR), m^−1^) for each site were obtained from Gardner et al. (2018).

### 2.2 Sample Collection

Coral fragments of ~2 cm from selected coral species (Table 3) were collected over a 2‐year period (2017–2018) from Bahrain and the Seychelles; Indonesian samples were collected in 2017 only. Sampling periods differed slightly among bioregions due to logistical and field constraints, which may influence direct regional comparisons. Common coral species were selected as they were found at all sites. Coral fragments came from different colonies with sampling depth between 5 and 10 m at each site; various numbers of replicates were collected based on availability at site (Table 3). Coral samples were collected randomly whilst ensuring a minimum of 1 m distance between sample colonies thereby randomizing host genetic effects and micro‐environments. The coral fragments were preserved in RNAlater (Invitrogen, Thermo Fisher Scientific) and placed on ice during transit, then at 3°C–6°C (in fridge) upon return from the field. Once samples arrived at the University of Essex they were stored at −20°C.

**TABLE 3:** List of coral species and total number of individual replicates (*n*).

Bahrain (AG)^a^	Seychelles (WIO)	Indonesia (CIP)
(April 2017 and 2018)	(May 2017 and 2018)	(July 2017)
*Porites lutea* (*n* = 16)	*Porites lutea* (*n* = 19)	*Porites lutea* (*n* = 9)
*Dipsastraea speciosa* (*n* = 2)	*Dipsastraea speciosa* (*n* = 17)	*Dipsastraea speciosa* (*n* = 10)
*Platygra daedalea* (*n* = 11)	*Favites pentagona* (*n* = 17)	*Favites pentagona* (*n* = 10)
*Cyphastrea microphtalma* (*n* = 10)	*Acropora muricata* (*n* = 13)	*Acropora muricata* (*n* = 10)
*Tubinaria peltata* (*n* = 3)	*Acropora gemmifera* (*n* = 8)	*Acropora gemmifera* (*n* = 10)
	*Pavona cactus* (*n* = 4)	*Pocillopora verrucosa* (*n* = 9)
		*Pocillopora damicornis* (*n* = 5)

^a^ NB: Samples were collected following the mass bleaching in Bahrain in 2017 which impacted the study reefs severely, making it very difficult to find live coral with signs of Symbiodiniaceae species presence for sampling. In 2018, samples were collected for *P. lutea* , *P. daedalea* , and *C. microphtalma* from Bahraini reefs.

### 2.3 Molecular Analysis

DNA was extracted from ~1 cm^2^ of the coral tissue on the skeleton from each sample (Table 3) using the Qiagen DNeasy Blood & Tissue Kit following the manufacturer's instructions with minor modifications (see Supporting Information 2). Symbiodiniaceae communities were characterized using a Nextera XT dual‐indexing strategy, which involved PCR amplification of a phylogenetic marker gene, followed by a secondary short‐cycle PCR amplification in which dual Nextera indices are added to the amplicon for multiplexing of samples. The ITS rRNA gene was targeted (~234–266 bp region) with Symbiodiniaceae specific primers SYM_VAR_5.8S2: (5′‐GAATTGCAGAACTCCGTGAACC‐3) and SYM_VAR_REV: (5′‐CGGGTTCWCTTGTYTGACTTCATGC‐3; Hume et al. 2018) both of which were modified to contain Illumina specific overhang sequences. The ITS rRNA gene was amplified in 25 μL reactions with 12.5 μL of appTAQ Polymerase (Appleton Woods LTD.), 1 μL of each primer (10 μM), 2 μL of Bovine Serum Albumin (BSA; 0.8 μg per reaction; Sigma Aldrich Co.), 6.5 μL PCR water and 2 μL template DNA. The PCR protocol included an initial denaturation step at 95°C for 3 min, followed by 35 cycles of 95°C for 30 s, 58°C for 30 s and 72°C for 30 s. After a final extension step of 72°C for 5 min, PCR products were held at 4°C. PCR products were purified using Agencourt AMPure XP PCR Purification beads (Beckman Coulter Ltd., High Wycombe, UK) following Illumina's “16S Metagenomic Sequencing Library Preparation” Protocol (https://bit.ly/1Ns3tAD).

The index PCR was carried out in 25 μL reactions with 12.5 μL of appTAQ Polymerase (Appleton Woods LTD.), 2.5 μL each of sample specific Series A & B Nextera XT index (Illumina), 5 μL PCR water (Bioline Reagents Ltd., UK) and 2.5 μL purified PCR product. PCR was conducted with an initial denaturation at 95°C for 3 min, followed by 8 cycles of 95°C for 30 s, 55°C for 30 s and 72°C for 30 s. A final extension step was included at 72°C for 5 min, after which PCR products were held at 4°C. PCR products were purified using Agencourt AMPure XP PCR Purification beads (Beckman Coulter Ltd., High Wycombe, UK) and quantified on a POLARstar Omega (BMG LABTECH GmbH, Germany) plate reader using the PicoGreen dsDNA assay. PCR products were then pooled in equimolar concentrations. The size and concentration of the resulting pool was checked twice, once using a NEBNext Library Quant Kit following the manufacturer's protocol (https://bit.ly/2Z0kmjf) on the BioRAD—CFXConnect Real Time System (96 well plate); and the second using the PicoGreen dsDNA assay on a NanoDrop 3300 Fluorospectrometer (Thermo fisher Scientific) following the manufacturer's protocol (https://bit.ly/2YY8iz5) to validate the concentration levels prior to sequencing. Next Generation Sequencing (NGS) was carried out on an Illumina MiSeq producing 2 × 301 bp sequences at the University of Essex, UK.

### 2.4 Bioinformatic Analysis

The generated NGS ITS raw sequence amplicon libraries were processed using the SymPortal analytical framework (https://symportal.org—described in Hume et al. 2019). Sequencing data was submitted to the SymPortal database for quality control (including the removal of non‐Symbiodiniaceae sequences) and analysis. Symbiodiniaceae genera were identified as part of the SymPortal analysis, which is conducted within a framework supported by a database containing sequencing data of each genus acquired from all previous run analyses cataloged within that database. The SymPortal database utilizes the sequence data submitted to it by global researchers to improve its ability to identify Symbiodiniaceae genotype‐representative ITS2 type profiles (hereafter referred to as ITS2 types) based on their presence and abundance in the samples and within the database. Unique combinations of intragenomic variations in sequences, commonly referred to as defining intragenomic [sequence] variants (DIVs), are used for profile characterization considering both sequence abundances and identities whereby sets of sequences found to re‐occur in multiple samples are searched for algorithmically (Howells et al. 2020). For more details regarding the SymPortal pipeline, quality control and algorithms used for ITS2 profile identification refer to Hume et al. (2019) and/or the accompanying GitHub site (https://github.com/didillysquat/SymPortal_framework; Hume et al. 2020). Of the 183 coral samples submitted for sequencing, 157 yielded Symbiodiniaceae sequences following SymPortal quality control and were retained for downstream analysis; the remaining 26 samples produced no assignable sequences and were excluded.

### 2.5 Data and Statistical Analysis

Symbiodiniaceae genera were analyzed using the output dataset derived from the SymPortal analysis to examine the symbiont community composition in different host species across bioregions. Analyses were performed using R (R Core Team 2023); all plots were created using the *ggplot* function in the “tidyverse” package (Wickham et al. 2019). ITS2 type abundance, relative abundance, ITS2 type richness (used here as a proxy for community richness), and diversity indices including Shannon's (*H*′) and Simpson's (1–*D*), which account for both abundance and evenness, were calculated using the *vegan* package in R (Oksanen et al. 2018). Analysis of variance (ANOVA) was used to test for significant difference in diversity metrics among bioregions and coral host species.

Principal coordinates analysis (PCoA) was used to explore the variation in ITS2 sequence profiles, based on the between‐profile and between‐sample distance matrices, generated from Symportal output for each coral genus (*Cladocopium* and *Durusdinium*; formerly, clade C and clade D respectively). To further examine potential drivers of the symbiont communities, variance partitioning in four cross factors was analyzed: coral host species, bioregion/site, thermal regime, and latitude. A permutational multivariate analysis of variance (PERMANOVA) was conducted to test for differences in Symbiodiniaceae community composition across bioregions within each coral host species (considered as a nested factor). Statistical significance was assessed using permutation‐based unadjusted *p*‐values (10,000 permutations) generated by the adonis2() function in the *vegan* package in R (Oksanen et al. 2018).

### 2.6 Limitations of Comparative Interpretation

This study was designed as a broad comparative assessment of coral‐associated Symbiodiniaceae communities across three distinct bioregions and thermal regimes. However, several limitations should be considered when interpreting comparative patterns among regions. Sampling effort and coral host representation were uneven across bioregions due to differences in coral availability, bleaching impacts, logistical constraints, and field accessibility. In addition, coral host species identifications were primarily based on colony‐level morphological characteristics following the taxonomic frameworks available at the time of sampling. Given recent advances in coral systematics and increasing evidence for cryptic diversity and geographically restricted species distributions across reef regions, some nominal coral species designations should be interpreted cautiously when making direct interregional comparisons. Biological sampling was conducted over different years among regions, which may influence direct regional comparisons due to interannual environmental variability.

Thermal regime characterization was standardized across all bioregions using long‐term SST metrics derived from the CoRTAD Version 6 database (1982–2017). These metrics were used to characterize broad regional thermal regimes rather than represent exact environmental conditions at the time of biological sampling. In addition, some environmental descriptors and site‐characterization variables were collected across different field expeditions and temporal periods as detailed in Table 2 while others, specifically the temperature and salinity ranges, were derived from published literature.

Furthermore, latitude, thermal regime, and site‐specific environmental characteristics are partially confounded within this dataset, limiting the ability to isolate the influence of individual variables independently. Consequently, the results presented here should be interpreted as exploratory biogeographical patterns intended to provide baseline insights into coral‐Symbiodiniaceae associations across contrasting reef systems.

## 3 Results

### 3.1 Overview of Symbiodiniaceae Composition Between Bioregions

**FIGURE 2:**
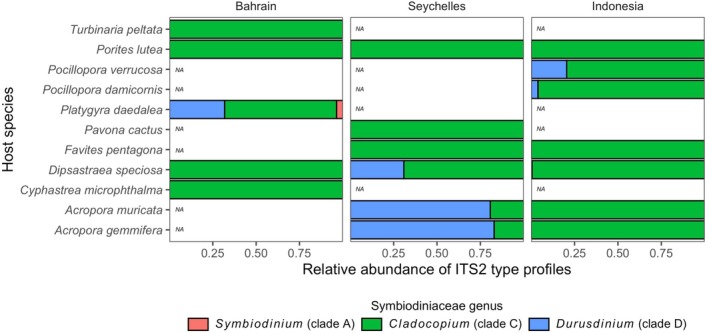
Relative abundance of Symbiodiniaceae genera (clades) based on ITS2 type profiles within each coral host species at sites in Bahrain (AG), Seychelles (WIO), and Indonesia (CIP). Individual coral colonies were sampled in both 2017 and 2018 in Bahrain and the Seychelles, whilst Indonesian coral colonies were sampled in 2017 only. NA = no samples acquired for that host species at a given site. The figure represents 157 coral colonies that yielded Symbiodiniaceae sequences following SymPortal quality control, out of 183 collected (see Table 3). The number of colonies retained per species per bioregion were: Bahrain: *Turbinaria peltata* (*n* = 3), *Porites lutea* (*n* = 16), *Platygyra daedalea* (*n* = 10), *Cyphastrea microphthalma* (*n* = 8), *Dipsastraea speciosa* (*n* = 1); Seychelles: *Porites lutea* (*n* = 19), *Pavona cactus* (*n* = 2), *Favites pentagona* (*n* = 8), *Dipsastraea speciosa* (*n* = 15), *Acropora muricata* (*n* = 13), *Acropora gemmifera* (*n* = 8); Indonesia: *Pocillopora verrucosa* (*n* = 9), *Pocillopora damicornis* (*n* = 4), *Porites lutea* (*n* = 9), *Favites pentagona* (*n* = 7), *Dipsastraea speciosa* (*n* = 9), *Acropora muricata* (*n* = 9), *Acropora gemmifera* (*n* = 7).

Amplicon sequencing generated 6,520,007 raw contigs across 157 samples that yielded sequences (range: 217–859,511 per sample). Following SymPortal quality control including chimera removal, taxonomic assignment and minimum entropy decomposition (Hume et al. 2019), 4,183,579 sequences were assigned to ITS2 type profiles representing three genera: *Cladocopium*, *Durusdinium* and *Symbiodinium* (Figure 2). Across all sampled sites/bioregions, the genus *Cladocopium* (corresponding clade C) was the most abundant endosymbiont accounting for 86.3% of sequences retained, followed by *Durusdinium* (corresponding clade D; 13.5%), while *Symbiodinium* (corresponding clade A) was observed to be the least abundant (0.2%) and detected only in one bioregion (AG).

*Cladocopium* was present in all 11 sampled host species whilst *Durusdinium* and *Symbiodinium* were found in seven and one host species, respectively (Figure 2; see Table 3 for full list of coral host species). Observed relative abundance and genus‐level associations of Symbiodiniaceae differed between bioregions and coral host species. In Bahrain (AG), which experiences extreme thermal conditions, all sampled coral colonies were associated with *Cladocopium* (100%), with the exception of *Platygyra daedalea* , which hosted a mixed symbiont community: *Cladocopium* (65%), *Durusdinium* (32%) and *Symbiodinium* (4%; Figure 2).

Conversely, most coral host species in the Seychelles (WIO) and Indonesia (CIP) exhibited co‐occurring associations with multiple Symbiodiniaceae genera. In the Seychelles, both *Favites pentagona* and *Pavona cactus* hosted solely *Cladocopium* (100%), whereas other coral species displayed mixed associations with both *Cladocopium* and *Durusdinium* (Figure 2). Within the *Acropora* genus ( *A. muricata* and *A. gemmifera* ), *Durusdinium* (81%) was more abundant in the Seychelles, while *Cladocopium* (19%) was present at lower levels.

In Indonesia, *F. pentagona* and *Porites lutea* exhibited exclusive associations with *Cladocopium* (100%), similar to their counterparts in other regions. However, the remaining coral host species, including *Acropora muricata* and *Pocillopora* spp., harbored both *Cladocopium* and *Durusdinium* (Figure 2). Notably, *A. muricata* in Indonesia was largely dominated by *Cladocopium* (99.98%) with trace presence of *Durusdinium* (0.02%), while *A. gemmifera* was associated solely with *Cladocopium* (100%; Figure 5).

*Symbiodinium* was only detected in Bahrain at a very low relative abundance (0.2%) and in a single coral host species ( *P. daedalea* ). Comparative analysis for this species across other bioregions was not possible due to lack of sample availability (Figure 2).

### 3.2 Symbiodiniaceae Diversity Across Bioregions

**FIGURE 3:**
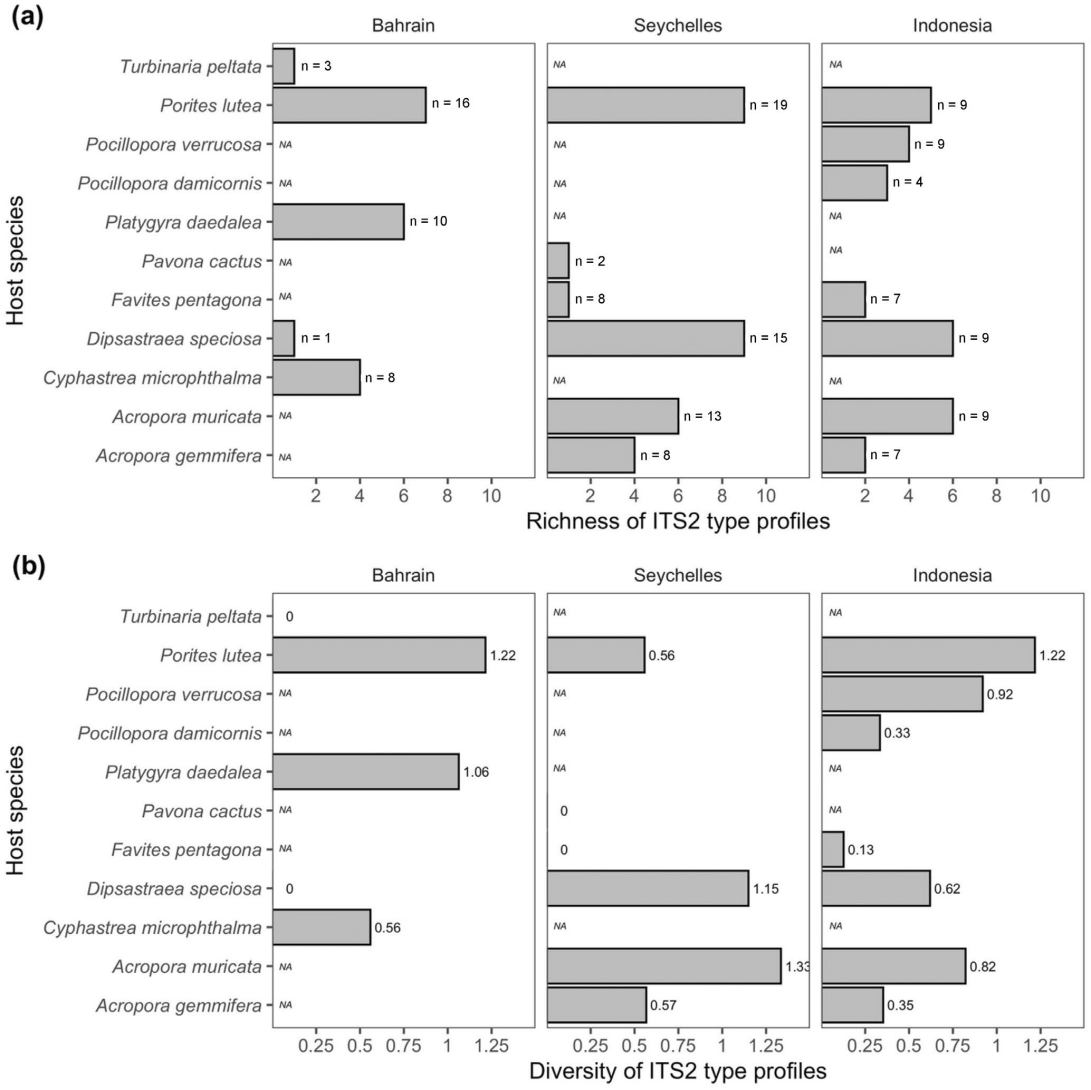
(a) Richness of coral associated‐Symbiodiniaceae ITS2 types across all sites in Bahrain (AG), Seychelles (WIO), and Indonesia (CIP). (b) Shannon's Diversity Index (*H*′) of coral associated‐Symbiodiniaceae ITS2 types across all sites (NA = no samples acquired from respective sites). Individual colonies were sampled in both 2017 and 2018 for Bahrain and the Seychelles, whilst Indonesian coral colonies were sampled in 2017 only.

Overall, a total of 50 distinct Symbiodiniaceae ITS2 types were identified across 11 coral host species in the three bioregions. However, analysis of variance (ANOVA) revealed no statistically significant differences in Symbiodiniaceae diversity across bioregions (ANOVA: *F*_2,17_ = 0.02, *p* = 0.98) or coral host species (ANOVA = *F*_10,17_ = 1.67, *p* = 0.26). Nevertheless, observed patterns indicated that in Bahrain, the highest diversity values were recorded in *P. lutea* (*H*′ = 1.2; 1–*D* = 0.7) and *P. daedalea* (*H*′ = 1.1; 1–*D* = 0.6), with lower diversity values in *Cyphastrea microphthalma* (*H*′ = 0.6; 1–*D* = 0.3), whilst *D. speciosa* , *T. peltata* , *P. cactus* , and *F. pentagona* each hosted a single ITS2 type (Figure 3b).

In the Seychelles, *A. muricata* exhibited the highest observed diversity values (*H*′ = 1.3; 1–*D* = 0.7), followed by *D. speciosa* (*H*′ = 1.2; 1–*D* = 0.6) and *P. lutea* (*H*′ = 0.6; 1–*D* = 0.3). *F. pentagona* and *P. cactus* each hosted a single ITS2 type (*H*′ = 0; 1–*D* = 0). In Indonesia, *P. lutea* again exhibited the highest diversity values (*H*′ = 1.2; 1–*D* = 0.7), followed by *A. muricata* and *D. speciosa* . In contrast, *F. pentagona* exhibited the lowest diversity (*H*′ = 0.1; 1–*D* = 0.1), while *A. gemmifera* exhibited moderate values (*H*′ = 0.4; 1–*D* = 0.2). Within the genus *Pocillopora* in Indonesia, *P. verrucosa* (*H*′ = 0.92; 1–D = 0.52) exhibited higher diversity values than *P. damicornis* (*H*′ = 0.33; 1–D = 0.20).

Across regions, the same host species exhibited varying degrees of diversity. For example, *P. lutea* in the Seychelles exhibited lower diversity values (*H*′ = 0.56; 1–D = 0.33) compared to *P. lutea* in Bahrain and Indonesia (both *H*′ = 1.22; 1–D = 0.70 and 0.67, respectively; Figure 3b). A similar descriptive pattern was seen in *Acropora*, where *A. muricata* exhibited higher diversity values than *A. gemmifera* in both the Seychelles and Indonesia (Figure 3b).

While these observed trends suggest differences in ITS2 type richness and diversity across bioregions and coral hosts, these patterns were not statistically significant and should therefore be interpreted with caution as exploratory descriptive patterns rather than definitive relationships.

### 3.3 Symbiodiniaceae Community Composition and Distribution Across Bioregions

**FIGURE 4:**
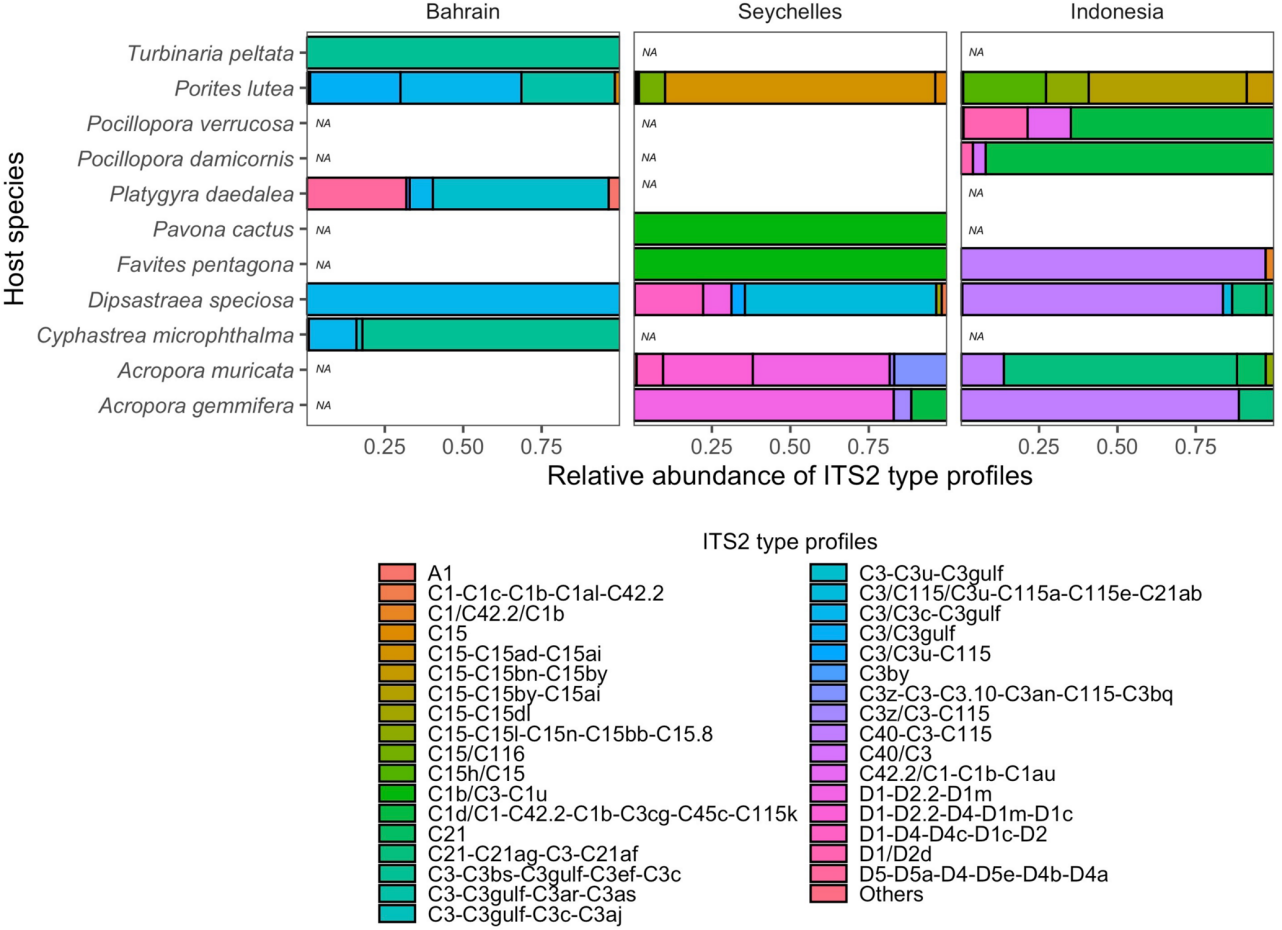
Relative abundance of Symbiodiniaceae ITS2 types within each coral host species across the three bioregions: Bahrain (AG), Seychelles (WIO), and Indonesia (CIP). Only ITS2 types with a relative abundance > 0.1% are shown. Coral colonies were sampled in both 2017 and 2018 for Bahrain and the Seychelles, whilst Indonesian colonies were sampled in 2017 only.

**FIGURE 5:**
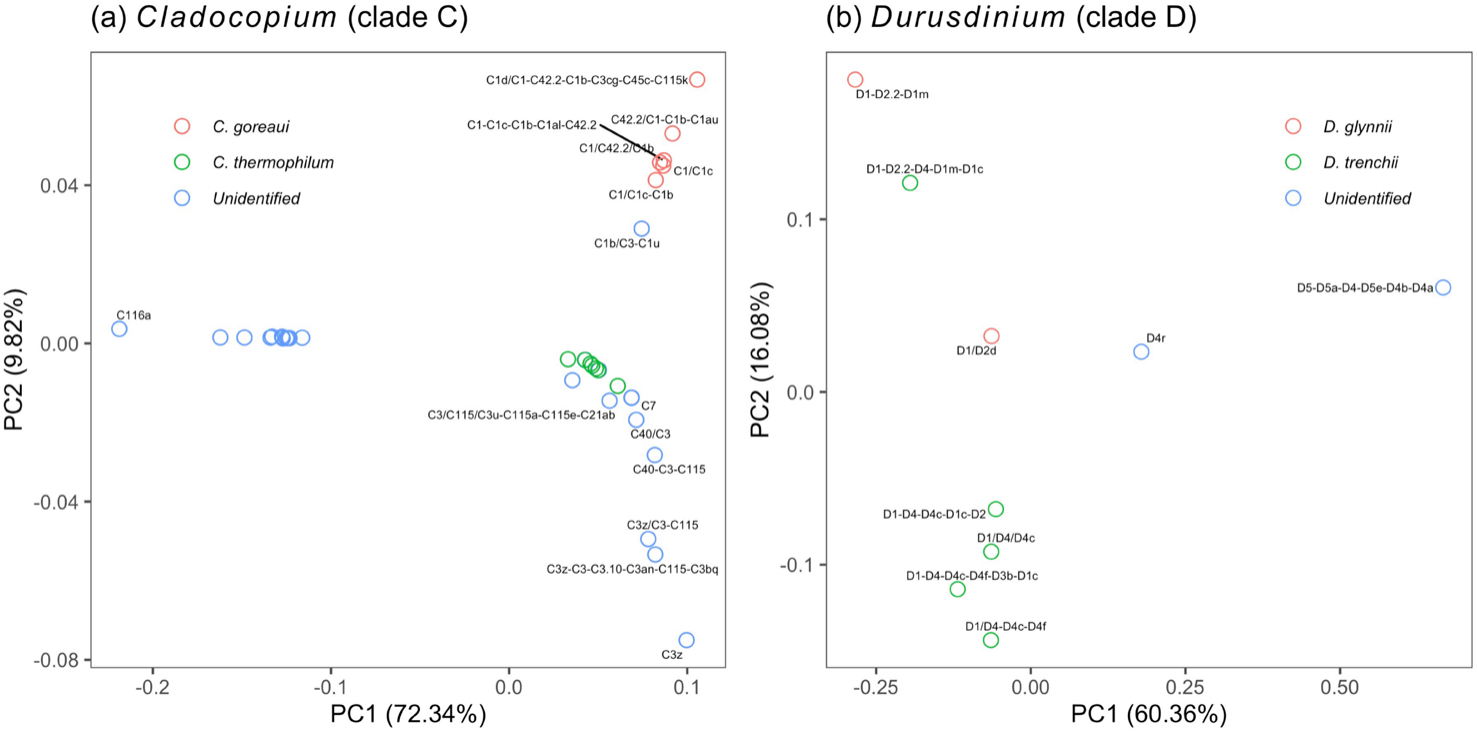
Principal coordinate analysis (PCoA) of Symbiodiniaceae ITS2 community composition across bioregions. (a) *Cladocopium* (clade C); (b) *Durusdinium* (clade D). Each point represents the Symbiodiniaceae community of an individual coral colony. Percentages on each axis indicate the proportion of variation explained by the respective coordinate axis.

Metabarcoding indicated that the relative abundance and distribution of Symbiodiniaceae ITS2 types varied across coral host species and bioregions. In total, 50 distinct ITS2 types were detected, with the highest number of types associated with *P. lutea* in the Seychelles (*n* = 9), and the lowest in *D. speciosa* in Bahrain (*n* = 1; Figure 4). However, it is important to note that many ITS type‐host associations were rare, occurring once or twice; therefore, only the most frequently occurring ITS2 types are visible in Figure 5.

In Bahrain, the Symbiodiniaceae community exhibited the lowest ITS type richness (*n* = 15). A notable feature of this region was the high relative abundance of the Gulf‐adapted type C3‐Gulf (*Cladocopium thermophilum*) which was detected in all sampled coral host species (Figure 4). *Porites lutea* hosted the greatest number of ITS2 types (7 out of 15), followed by *P. daedalea* (6 types), and *C. microphtalma* (4 types; Figure 4). In contrast, both *D. speciosa* and *T. peltata* hosted only a single ITS2 type each. Overall, C3‐Gulf was the most abundant ITS2 type within the Bahrain samples, with other notable contributions from C15, D1 (*Durusdinium glynnii*), and D5.

The Seychelles hosted an intermediate number of ITS2 types (*n* = 18) whilst *P. lutea* and *D. speciosa* were each associated with nine ITS2 types spanning both *Cladocopium* and *Durusdinium*. *D. speciosa* in the Seychelles also hosted the typically Gulf‐associated type C3‐Gulf, although detected only at trace relative abundance (0.1%; Figure 5; Supporting Information 3, Table S3.1). Meanwhile, *P. cactus* and *F. pentagona* each were observed to host a single ITS2 type. The most abundant ITS2 types across Seychelles samples were C3, C1 and C15, with a notable presence of D1 (*Durusdinium glynnii*).

Indonesia exhibited the highest ITS2 type richness (19 types) among the three study bioregions. *A. muricata* and *D. speciosa* hosted the greatest number of ITS2 types i.e., 6 types each, while *A. gemmifera* exhibited the lowest richness within the genus *Acropora*, associating with only two *Cladocopium* ITS2 types (Figure 4). A similar pattern of lower ITS2 type richness in *A. gemmifera* relative to *A. muricata* was also observed in the Seychelles. The most abundant ITS2 types in Indonesia included C15, C21, C2, and D4.

Differences in community composition were not only observed across regions but also within coral host genera. For example, while *A. muricata* consistently harbored a greater diversity of ITS2 types across Indonesia and the Seychelles, *A. gemmifera* exhibited lower richness in both regions (Figure 4).

**FIGURE 6:**
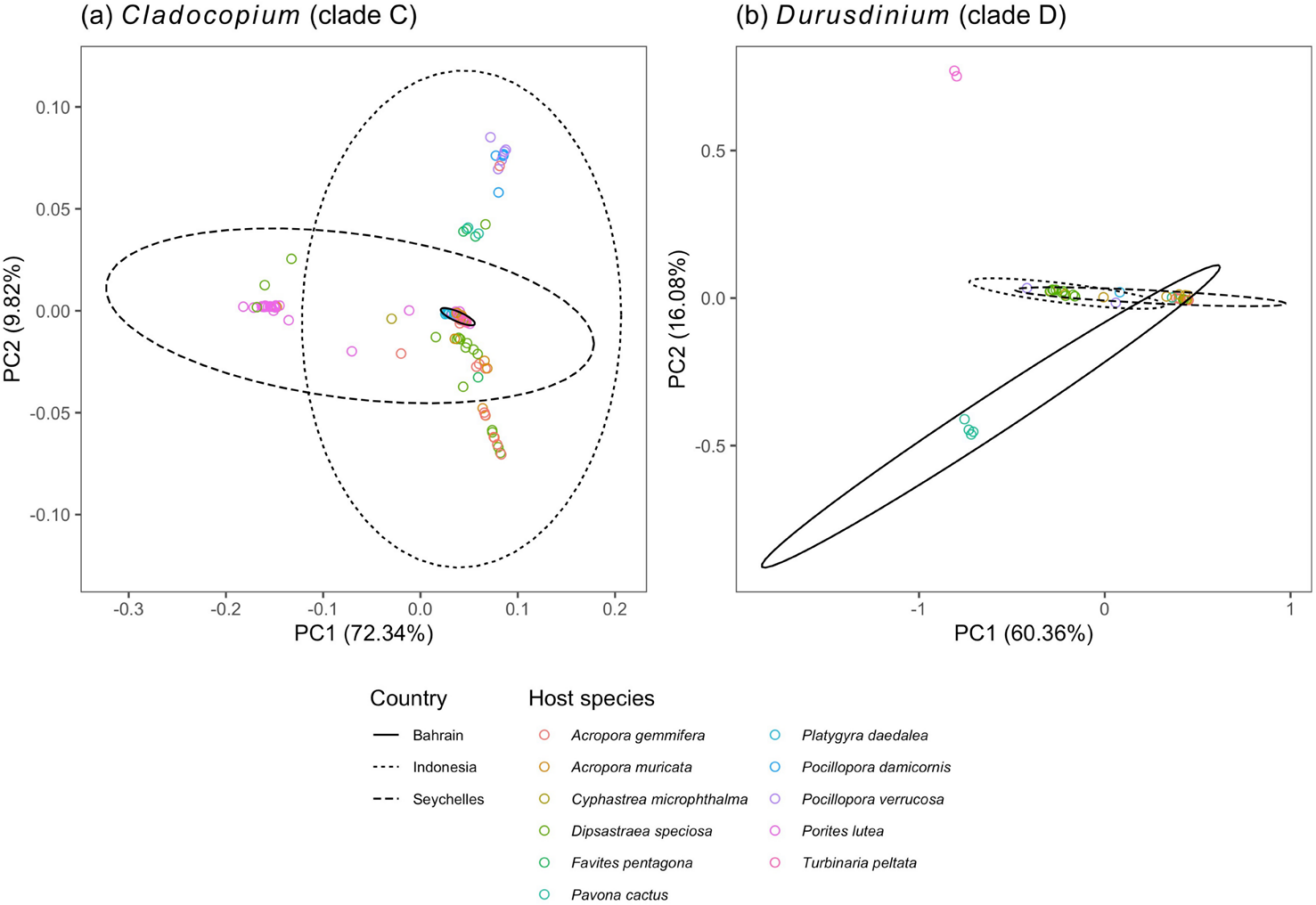
Principal coordinate analysis (PCoA) of Symbiodiniaceae ITS2 community composition by coral host species and bioregion: Bahrain (AG), Seychelles (WIO), and Indonesia (CIP). (a) *Cladocopium* (clade C); (b) *Durusdinium* (clade D). PERMANOVA results (Adonis *p* values) indicate significant differences in community structure and are detailed in Supporting Information 3, Table S3.2.

Our principal component analysis (PCoA) showed clustering patterns in Symbiodiniaceae communities according to coral host species, thermal regime, and geographic positioning (Figure 6). These patterns were further supported by PERMANOVA, which showed that community composition differed significantly according to coral host species (*p* < 0.001), thermal regime (*p* < 0.01), site (*p* < 0.01), and latitude (*p* < 0.05) for both *Cladocopium* and *Durusdinium* lineages (Supporting Information 3, Table S3.2). Collectively, these results suggest that coral host species, thermal regime, and geographic setting may contribute to variation in Symbiodiniaceae community composition (Figure 6). However, these variables are partially confounded within the present dataset and should therefore not be interpreted as independent drivers.

Lastly, *Cladocopium* was observed to be the most diverse genus across all bioregions, although many ITS types within this group remain unidentified and undescribed (Figure 5), suggesting the presence of additional Symbiodiniaceae diversity yet to be characterized.

## 4 Discussion

This study explored the diversity and distribution of coral‐Symbiodiniaceae associations across multiple coral host species spanning three distinct bioregions, each characterized by differing thermal regimes and latitudinal gradients. Using next‐generation sequencing of the ITS2 region, this study provides the first ITS2‐based molecular characterization of Symbiodiniaceae communities in Bahrain and documents the presence of *Cladocopium thermophilum* (C3‐Gulf) across all sampled coral host species in the region, with the exception of *P. daedalea* which hosted a mixed symbiont assemblage. The high relative abundance of this thermotolerant ITS2 type within Bahraini samples may reflect the extreme thermal conditions characteristic of reefs in the region.

In addition, our results showcased a 50% reduction in the relative abundance of the common generalist *Cladocopium* (C3) in the Seychelles compared to Indonesia. Although *Cladocopium* types are typically dominant in coral assemblages under moderate thermal conditions, previous studies have suggested that some members of this group may be sensitive to elevated temperatures (Jones and Berkelmans 2010). This observed pattern may therefore reflect the prolonged thermal stress experienced by reefs in the Seychelles during the 2016 global bleaching event (DHW = 7°C‐weeks), relative to the comparatively lower thermal stress recorded in Indonesia (DHW = 5°C‐weeks). While these observations should be interpreted cautiously, they are consistent with previous studies (e.g., Terraneo et al. 2024; Chan et al. 2023; Cunning et al. 2015) suggesting that bleaching events and thermal stress may influence Symbiodiniaceae community composition by favoring more thermotolerant symbiont types in reefs exposed to more frequent or intense heat stress.

### 4.1 Shifts in Symbiodiniaceae Diversity and Abundance Across Three Bioregions

Overall, three distinct genera (i.e., *Cladocopium*, *Durusdinium*, and *Symbiodinium*) of coral‐Symbiodiniaceae associations were found across all sampled sites. In general, *Cladocopium* (clade C) was the most abundant whilst *Durusdinium* (clade D) was observed to co‐occur across the three bioregions as seen in our sequencing data, which showed *Cladocopium* accounting for 86.3% of relative read abundance across samples.

Prevalence of *Cladocopium* has been reported as a common feature of Symbiodiniaceae communities in the Arabian Gulf (Ziegler et al. 2017; Howells et al. 2020), Central Indo‐Pacific (LaJeunesse 2005; Thomas et al. 2014), and Western Indian Ocean (Gardner et al. 2018; Leveque et al. 2019). *Cladocopium* is usually reported as the dominant type when water temperatures remain below the bleaching threshold (Smith et al. 2017). Previous studies have documented the occurrence of *Cladocopium goreaui* (C1) and ITS2 type C3 worldwide; both of which are considered generalist types (LaJeunesse et al. 2004; LaJeunesse 2005; Leveque et al. 2019). Observed patterns in the present study are consistent with previous reports describing shifts in C3 prevalence across reefs spanning different latitudinal and thermal regimes, whereby the commonly observed low latitude C3 types become less prevalent or absent on high‐latitude reef environment (LaJeunesse et al. 2004). Within our dataset, this pattern was reflected by a shift from the common C3 generalist genotypes (e.g., C3/C115, C3z/C3‐C115, C3/C34‐C115) observed in the Seychelles and/or Indonesia sites toward the specialized differentiated monophyletic lineage, C3‐Gulf (*Cladocopium thermophilum*; Hume et al. 2015) in Bahrain. While these observations should be interpreted cautiously given the exploratory nature of the dataset, they are consistent with previous studies suggesting that regionally endemic symbiont types may play an important role in coral persistence under extreme thermal conditions. The ITS2 C3‐Gulf type has previously been associated with increased coral tolerance under the extreme summer temperatures (~36**°**C) that the reefs in the Gulf are exposed to (D'Angelo et al. 2015).

*Durusdinium glynnii* (D1) was associated with coral host species in Bahrain and the Seychelles. *Durusdinium* symbionts are generally considered stress/thermally tolerant (Stat et al. 2013); this lineage is observed to contribute notably to the relative abundance and richness of ITS2 types in the Seychelles. Furthermore, *Durusdinium trenchii* is often associated with recently bleached and/or recovering corals (Baker 2001; Baker et al. 2004), which can be replaced or outcompeted through the process of “shuffling” following recovery (Thornhill et al. 2006; Baumann et al. 2018). This association in warmer thermal regimes may reflect the aftermath of a site‐specific bleaching event which occurred in the Seychelles in 2016 (Gardner et al. 2018) and reported in other regions (Kemp et al. 2014; Smith et al. 2017). Previous studies have suggested that hosting these stress tolerant symbionts can increase coral's thermal tolerance by 1.0°C–2.0°C (Stat and Gates 2011) when compared to the thermally sensitive *Cladocopium* generalist (C3 type; Berkelmans and Van Oppen 2006). However, this enhanced resilience caused by representatives from the *Durusdinium* type comes at an energetic cost to the coral host, impacting their growth and accretion rate (Baker 2001; LaJeunesse et al. 2009; Pettay et al. 2015). This trade‐off highlights how environmental pressures may shape symbiotic assemblages differently depending on the ecological context.

Furthermore, the genus *Symbiodinium* (clade A) was observed to occur in the high latitude sites of Bahrain which mirrors previous reports describing its occurrence in higher latitude reef environments (Savage et al. 2002; Baker 2003; Leveque et al. 2019). *Symbiodinium* associates are characterized as high temperature specialists with high irradiance (LaJeunesse 2002). Previous studies have demonstrated that numerous *Symbiodinium* associates are tolerant to high light and temperature whilst being facilitated by enhanced photo‐acclimation and photo‐protective pathways (Robison and Warner 2006; Reynolds et al. 2008; Takahashi et al. 2009; Kemp et al. 2014). In our present study, *Symbiodinium* was detected in *P. daedalea* (a common thermal stress tolerant coral in AG; Kirk et al. 2018) in Bahrain which, among the coral species, exhibited one of the most diverse and richest symbiont assemblages (C = 65%, D = 32% and A = 4%). This multi‐lineage association may reflect both the symbiotic flexibility of *P. daedalea* and the environmental conditions characteristic of Bahrain's extreme thermal regime.

Out of the three bioregions, Indonesia had the highest richness and relative abundance of ITS2 types. Located in the Coral Triangle, Indonesia is not only the epicenter of coral diversity; it is also considered the center of Symbiodiniaceae ITS2 genotype diversity (LaJeunesse et al. 2012; Chen et al. 2019), hence results are in line with previous observations. Our results suggest that Indonesia harbored the greatest number of distinct ITS2 types among the sampled bioregions. This is likely influenced by the intermediate disturbance hypothesis, which states that ecosystems are more stable and diverse when disturbances occur at intermediate levels of frequency and intensity.

Indonesian corals host a mixture of *Cladocopium* types, some of which are thermally sensitive generalists (e.g., C3) whilst others are more thermally tolerant (e.g., C15; Pochon et al. 2004). Moreover, observations of symbiont types within *P. verrucosa* and *P. damicornis* in Indonesia support those made by other studies reporting C1 is the most prevalent ITS2 type (LaJeunesse et al. 2004; Yang et al. 2012). Although previous studies reported the detection of C1c within *Pocillopora* sp. in the Pacific (Liang et al. 2021; Quigley et al. 2017), we detected ITS2 C1d type. This intra‐genus variability may reflect either host genotype effects and/or localized symbiont pool availability. In addition, it remains unclear as to why Symbiodiniaceae assemblages differ in diversity between *A. muricata* and *A. gemmifera* despite belonging to the same genus and being collected from the same site/bioregion and thermal regime. One suggestion is that these differences may be related to heritability and whether symbionts were horizontally (acquired from the environment) or vertically (passed on maternally) transferred (Quigley et al. 2017). Exploring this further through larval rearing experiments or host‐symbiont genotype matching could provide deeper insight.

Overall, observed patterns in our present study suggest lower diversity and relative abundance of ITS2 types in the higher latitude and more thermally extreme reefs in Bahrain compared to the lower latitude reefs of Indonesia and the Seychelles. Similar broad‐scale biogeographical patterns have previously been reported by Chen et al. (2019). In addition, Symbiodiniaceae community composition in Bahrain was comparatively even, with C3‐Gulf representing the most abundant ITS2 type across all sampled coral species, whereas more compositionally variable Symbiodiniaceae communities were observed among coral species in Indonesia and the Seychelles. While these observations should be interpreted cautiously given the partial confounding among latitude, site and thermal regime within the dataset, the observed patterns may reflect differences in environmental conditions and geographic separation among the study regions. The relative uniformity observed within Bahrain's symbiont profile may be consistent with environmental filtering under chronic thermal stress.

Chen et al. (2019) suggested that geographical distance could limit the spread of Symbiodiniaceae species since evidence indicates that many free‐living Symbiodiniaceae have an in situ life span of approximately 7 days (Nitschke 2015). Their short life‐span and limited ability to swim short distances (3–10 m/day; Fitt and Trench 1983), combined with dependence on sea currents for their dispersal (Wirshing et al. 2013; Thornhill et al. 2017; Chen et al. 2019), may contribute to broad‐scale differences in Symbiodiniaceae diversity and richness across reef systems (Chen et al. 2019). These factors (i.e., currents, geographical distance, short‐life span) together with environmental stressors such as extreme thermal regimes and high salinity levels in the Arabian Gulf, have previously been suggested to influence Symbiodiniaceae diversity, distribution and host associations (D'Angelo et al. 2015; Ziegler et al. 2017; Smith et al. 2017).

### 4.2 Community Composition and Biogeographical Patterns of Symbiodiniaceae

The Symbiodiniaceae community composition in the Seychelles was distinct and featured a notable contribution from thermally tolerant *Durusdinium* types, despite the continued prevalence of thermally sensitive *Cladocopium* lineages.

One particularly notable finding was the detection of the C3‐Gulf ITS2 type (*Cladocopium thermophilum*) in *D. speciosa* in the Seychelles, although only at a trace relative abundance (0.1%). While C3‐Gulf has previously been confirmed in the AG (Hume et al. 2015; D'Angelo et al. 2015), where it is associated with extreme thermal regimes, and has largely been considered regionally restricted, the present study, to our knowledge, documents its molecular detection within a coral sample from the WIO. Although detected at a very low abundance, this finding remains biogeographically noteworthy as it suggests that the distribution of this thermotolerant ITS2 type may extend beyond its previously recognized range. However, given the low relative abundance observed, the significance, persistence, and functional role of this association remain uncertain and should therefore be interpreted cautiously. In addition, contamination or transient/background occurrence cannot be fully excluded and further independent extractions, sequencing, and targeted regional sampling would be required to verify the persistence and ecological relevance of this detection. Nevertheless, this detection raises important questions regarding the dispersal, connectivity, and potential cryptic distribution of thermotolerant Symbiodiniaceae lineages across reef systems, highlighting the need for further targeted sampling and molecular investigation across the wider WIO region.

Suggestions have been made that the Gulf of Oman could play a role in facilitating the adaptation of corals in the Indian Ocean to rising planetary temperatures by providing a potential source of these thermotolerant symbionts (D'Angelo et al. 2015). Furthermore, previous studies have suggested that Symbiodiniaceae species composition in Bahrain and the wider AG may shift from thermally sensitive ITS2 types (e.g., common generalist *Cladocopium*) toward more thermally tolerant types (e.g., *Durusdinium*) as a mechanism to adapt and acclimatize to long‐term exposure to elevated temperatures (Berkelmans and Van Oppen 2006; Palumbi et al. 2014; Wang et al. 2019).

The detection of C3‐Gulf in a thermally stressed region such as the Seychelles raises the possibility that thermotolerant genotypes may occur beyond their previously recognized range. Whether this represents ongoing gene flow, cryptic historical presence, transient background occurrence, or broader biogeographical connectivity remains unclear, but it raises important questions regarding the evolutionary and ecological plasticity of these symbionts. Moreover, it invites further investigation into the regional current systems, coral larval dispersal, and symbiont migration patterns that could facilitate such events.

Observed patterns from our study also suggest a potential shift in *Symbiodiniaceae* species composition under thermally stressful conditions, particularly in Bahrain, where long‐term exposure to elevated sea surface temperatures and hypersalinity was associated with a high relative abundance of thermotolerant ITS types such as C3‐Gulf and *Durusdinium glynnii*. Similar patterns were observed in the Seychelles, which experienced severe bleaching in 2016 (DHW = 7°C‐weeks), where *Durusdinium* contributed notably to the Symbiodiniaceae assemblages observed across several coral hosts. While these observations should be interpreted cautiously, they are consistent with previous studies describing symbiont “shuffling” and adaptive shifts in coral symbiosis under environmental stress (Berkelmans and Van Oppen 2006; Palumbi et al. 2014), where coral holobionts may alter their resident symbiont communities in response to changing environmental conditions.

In contrast, reefs in Indonesia, which experienced comparatively milder thermal stress (DHW = 5°C‐weeks in 2016), hosted the most taxonomically diverse *Symbiodiniaceae* assemblages. This richness, dominated by *Cladocopium* ITS2 types including C1, C3, and C15, appears to reflect both stable environmental conditions and the high biodiversity of the Coral Triangle.

The absence of highly stress‐tolerant types such as C3‐Gulf in Indonesia and their trace occurrence in the Seychelles may further suggest localized ecological filtering and biogeographical boundaries influencing Symbiodiniaceae distribution patterns (LaJeunesse et al. 2012; Chen et al. 2019).

Together, these observations suggest that Symbiodiniaceae community composition varies across thermal regime, geographic regions, and historical disturbance, with thermotolerant ITS2 types such as C3‐Gulf potentially representing important components of symbiont assemblages associated with environmentally extreme reef systems.

## 5 Conclusion

In conclusion, observed biogeographical patterns of Symbiodiniaceae diversity, distribution, and community composition varied across the environmental conditions, thermal regimes, and latitudinal gradients represented in our study. This study presents the first molecular characterization of Symbiodiniaceae ITS2 types in Bahrain, home to some of the world's hottest coral reefs and provides molecular evidence for the presence of the thermotolerant C3‐Gulf (*Cladocopium thermophilum*) type in the region based on next‐generation sequencing data. In addition, this work documents the detection of the C3‐Gulf type in the Seychelles, extending previous observations beyond the AG and the Gulf of Oman, although the ecological significance of this low‐abundance detection remains uncertain and should be interpreted cautiously.

Findings from Indonesia further support earlier observations that reefs in lower latitudes and under comparatively moderate thermal regimes may host more taxonomically rich and diverse assemblages of Symbiodiniaceae types across coral species. Together, these results contribute to a broader understanding of coral–Symbiodiniaceae associations across contrasting reef systems, particularly in relation to how symbiont communities may shift or respond under different thermal and biogeographical contexts. In light of a rapidly changing climate, these findings provide important baseline insights that may help inform future research into coral resilience, symbiont flexibility, and reef conservation strategies.

## Author Contributions

**Reem K. AlMealla:** conceptualization (equal), data curation (lead), formal analysis (equal), funding acquisition (equal), investigation (lead), methodology (equal), project administration (lead), resources (equal), validation (equal), visualization (equal), writing – original draft (lead), writing – review and editing (supporting). **Brisneve Edullantes:** data curation (equal), formal analysis (equal), visualization (equal), writing – original draft (supporting), writing – review and editing (equal). **Boyd McKew:** data curation (equal), formal analysis (supporting), resources (equal), software (lead), writing – review and editing (equal). **Kirsty Matthews Nicholass:** investigation (supporting), writing – review and editing (equal). **Leanne J. Hepburn:** conceptualization (equal), methodology (supporting), project administration (supporting), resources (equal), supervision (equal), writing – review and editing (equal). **Bethan Greenwood:** investigation (supporting), resources (supporting), writing – review and editing (equal). **David J. Smith:** conceptualization (equal), funding acquisition (equal), investigation (supporting), methodology (supporting), resources (equal), supervision (equal), writing – review and editing (equal). **Jamaluddin Jompa Adalah:** writing – review and editing (equal). **Gilberte Gendron:** writing – review and editing (equal). **Michelle L. Taylor:** conceptualization (equal), investigation (equal), methodology (equal), resources (equal), supervision (lead), writing – original draft (supporting), writing – review and editing (equal).

## Funding

Funding for this study's molecular work and Bahrain field work was provided by Gulf Petrochemical Industries Co. (BSC). Grant/Award Number: GPIC/P/D/28/2018. Funding to execute fieldwork in the Seychelles was provided by Mitsubishi Motors (Mitsubishi Corporation) and Earthwatch Institute. Funding to execute field work in Indonesia was provided by Operation Wallacea.

## Disclosure

Benefit‐Sharing Statement: This research was conducted in full compliance with national regulations in each country where samples were collected. All necessary research and collection permits were obtained from the relevant governmental authorities: the Supreme Council for Environment (Bahrain), the Seychelles Parks and Garden Authority (formerly SNPA), and the Indonesian Ministry of Research, Technology and Higher Education (RISTEK). Collaborative partnerships were established with scientists and institutions from each provider country, and these collaborators are included as co‐authors on this manuscript. Research results have been shared with national partners and contribute directly to regional biodiversity knowledge and coral reef conservation efforts. The Nagoya Protocol was not applicable to this work; however, equitable scientific collaboration and transparent data sharing have been upheld, and all data will be made publicly accessible via the NCBI repositories described above.

## Conflicts of Interest

The authors declare no conflicts of interest.

## Supporting information


**Supporting Information 1:** Symbiodiniaceae genera, assigned clades/types and species.
**Table S1:** Symbiodiniaceae genera, assigned clades/types and species (*lacks ITS alphanumeric designation‐could be a different/new species).
**Supporting Information 2:** Symbiodiniaceae DNA extraction protocol and relative abundance of ITS2 types.
**Supporting Information 3:** Supporting Information on Symbiodiniaceae community composition and statistical analyses across bioregions.
**Table S3.1:** Relative abundance of ITS2 type profiles across coral host species and bioregions. The row highlighted in red indicates the novel detection of the C3‐Gulf ITS2 type in the Seychelles.
**Table S3.2:** Results of PERMANOVA on Principal Coordinate Analysis (PCoA) of Symbiodiniaceae ITS2 community composition across coral host species, thermal regimes, individual sites, and latitudinal transects. Analyses were conducted separately for *Cladocopium* (clade C) and *Durusdinium* (clade D). Significant *p*‐values (*p* < 0.05) are marked in (*), (*p* < 0.001) are marked in (**) and (*p* < 0) are marked in (***).

## Data Availability

Raw ITS2 sequence reads have been deposited in the NCBI Sequence Read Archive (SRA) under BioProject accession number PRJNA1377275. All samples and their associated metadata (coral host species, bioregion, site information, and sampling year) are archived through the linked BioSample accessions. Symbiodiniaceae ITS2 type profiles generated via SymPortal, along with additional methodological details and relative abundance tables are provided in the Supporting Information.

## Endnotes

^1^
Coral Reef Temperature Anomaly Database (CoRTAD) is a dataset developed for coral reef ecosystem applications by NOAA and embodies thermal stress metrics. The CoRTAD Version 6 database used in this study contains global ~4 km resolution SST data on a weekly time scale for the period 1982–2017. ftp://ftp.nodc.noaa.gov/pub/data.nodc/cortad/README.html.
