# Supplementary material for: Biogeographical Patterns of Coral‐Associated Symbiodiniaceae Across Thermal Regimes Including Trace Molecular Detection of Cladocopium thermophilum (C3‐Gulf) in the Seychelles
Source: Ecol Evol. 2026 Jul 26;16(7):e73941. doi: 10.1002/ece3.73941 (PMC13402104; doi:10.1002/ece3.73941)
Supplement: Supplementary file 1 — Supporting Information 1: Symbiodiniaceae genera, assigned clades/types and species. Table S1: Symbiodiniaceae genera, assigned clades/types and species (*lacks ITS alphanumeric designation‐could be a different/new species). Supporting Information 2: Symbiodiniaceae DNA extraction protocol and relative abundance of ITS2 types. Supporting Information 3: Supporting Information on Symbiodiniaceae community composition and statistical analyses across bioregions. Table S3.1: Relative abundance of ITS2 type profiles across coral host species and bioregions. The row highlighted in red indicates the novel detection of the C3‐Gulf ITS2 type in the Seychelles. Table S3.2: Results of PERMANOVA on Principal Coordinate Analysis (PCoA) of Symbiodiniaceae ITS2 community composition across coral host species, thermal regimes, individual sites, and latitudinal transects. Analyses were conducted separately for Cladocopium (clade C) and Durusdinium (clade D). Significant p‐values (p < 0.05) are marked in (*), (p < 0.001) are marked in (**) and (p < 0) are marked in (***). [file ECE3-16-e73941-s001.docx]

***Supplementary Material***

**Article Title: Biogeographical Patterns of Coral-Associated Symbiodiniaceae Across Thermal Regimes Including Trace Molecular Detection of of *Cladocopium thermophilum* (C3-Gulf) in the Seychelles**

Reem K. AlMealla^1,2*^, Brisneve Edullantes^1,3^ , Boyd McKew^1^, Kirsty Matthews Nicholass^4^, Leanne J. Hepburn^1^, Bethan Greenwood^1^, David J. Smith^1^, Jamaluddin Jompa^5^, Gilberte Gendron^6,7^, Michelle L. Taylor^1^

^1^School of Life Sciences, University of Essex, Colchester, Essex, UK

^2^Nuwat for Environmental Research & Education, Al Janabiyah, Kingdom of Bahrain

^3^Department of Biology and Environmental Science, College of Science, University of Philippines Cebu, Lahug, Cebu, Philippines 6000

^4^School of Biological and Marine Sciences, University of Plymouth, UK

^5^Hasanuddin University, Department of Fisheries, Makassar, Sulawesi, Indonesia

^6^ Island Biodiversity and Conservation Centre, University of Seychelles

^7^ Bee Ecological Consulting, Victoria, Seychelles

***Corresponding Author**: Reem K. AlMealla (email: reem@nuwat.org)

**Supplementary Material 1.** Symbiodiniaceae genera, assigned clades/types and species

**Table S1.** Symbiodiniaceae genera, assigned clades/types and species (*lacks ITS alphanumeric designation-could be a different/new species).

| **Clade/Type** | **Species** | **Source** |
| --- | --- | --- |
| A | *Symbiodinium sp.* | LaJeunesse *et al.*, 2018 |
| A* | *Symbiodinium natans* | Hansen & Daugbjerg, 2009 |
| A1/A1.1 | *Symbiodinium microadriaticum* | Lee *et al.*, 2015 |
| A2 | *Symbiodinium pilosum* | Lee *et al.*, 2015 |
| A3/A3^Pacific^/A3a/  A3x/A6 | *Symbiodinium tridacnidorum* | Lee *et al.*, 2015 |
| A4 | *Symbiodinium linuchae* | Trench & Thinh, 1995 |
| A13/A1.1 | *Symbiodinium necroappetens* | LaJeunesse *et al.*, 2015 |
| B | *Breviolum sp.* | LaJeunesse *et al.*, 2018 |
| B* | *Breviolum aenigmaticum* | Parkinson *et al.*, 2015 |
| B1 | *Breviolum antillogorgium* | Parkinson *et al.*, 2015 |
| B1 | *Breviolum minutum* | Lajeunesse *et al.*, 2012 |
| B1 | *Breviolum pseudominutum* | Parkinson *et al.*, 2015 |
| B2 | *Breviolum psygmophilum* | Lajeunesse *et al.*, 2012 |
| B7 | *Breviolum endomadracis* | Parkinson *et al.*, 2015 |
| C | *Cladocopium sp.* | LaJeunesse *et al.*, 2018 |
| C1 | *Cladocopium goreaui* | Davies *et al.*, 2018 |
| C3-Gulf | *Cladocopium thermophilum* | Hume *et al.*, 2015 |
| D | *Durusdinium sp.* | LaJeunesse *et al.*, 2018 |
| D1 | *Durusdinium glynnii* | Wham *et al.*, 2017 |
| D1a | *Durusdinium trenchii* | Davies *et al.*, 2018 |
| D15 | *Durusdinium boreum* | LaJeunesse *et al.*, 2014 |
| D8/D12-13/D13 | *Durusdinium eurythalpos* | LaJeunesse *et al.*, 2014 |
| E1 | *Effrenium voratum* | Jeong *et al.*, 2014 |
| F1 | *Fugacium kawagutii* | Trench, 2000 |
| G/G3/G3.3 | *Gerakladium sp.* | LaJeunesse *et al.*, 2018 |
| G* | *Gerakladium spongiolum* | Ramsby *et al.*, 2017 |
| G* | *Gerakladium endoclionum* | Ramsby *et al.*, 2017 |

**Supplementary Material 2.** Symbiodiniaceae DNA Extraction Protocol and Relative Abundance of ITS2 Types

### S2 | Qiagen DNeasy Blood & Tissue Kit – Protocol Method Modification

- Add 180 μl of ATL Buffer to a 1.5 ml micro-centrifuge tube.
- Place coral tissue into the micro-centrifuge tube, grind coral tissue sample in the tube to mix with ATL Buffer.
- Add 30 μl of proteinase K to the sample and vortex.
- Incubate in thermomixer at 56°C overnight at 400 rpm.
- Add 4 μl of RNAase A and leave to incubate at room temperature for 2 minutes
- Add 200 μl of AL Buffer and vortex.
- Add 200 μl of 100% ethanol, vortex and centrifuge.
- Pipette mixture into spin column in 2 ml collection tube. Centrifuge at 8000 rpm for 1 min. Discard flow through and collection tube.
- Place spin column into new 2 ml collection tube and add 500 μl of AW1 Buffer. Centrifuge at 8000 rpm for 1 minute. Discard flow through and collection tube.
- Place spin column into new 2ml collection tube and add 500 μl of AW2 Buffer. Centrifuge for 3 minutes at 14000 rpm. Discard flow through and collection tube.
- Transfer spin column to new 1.5 / 2 ml micro-centrifuge tube.
- Elute the DNA by adding 70 μl AE Buffer to center of spin column membrane, incubate for 5 mins at room temperature then centrifuge for 1 minute at 8000 rpm.
- Pipette solution from the micro-centrifuge tube back into spin column, incubate for 5 mins at room temperature then centrifuge for 1 minute at 8000 rpm.
- Discard of filter top and spin column.
- DNA is now in the bottom of the 1.5 μl micro-centrifuge tube ready for use.

**Supplementary Material 3**: Supplementary Data on Symbiodiniaceae Community Composition and Statistical Analyses Across Bioregions

**Table S3.1.** Relative abundance of ITS2 type profiles across coral host species and bioregions. The row highlighted in red indicates the novel detection of the C3-Gulf ITS2 type in the Seychelles.

| **Species** | **ITS2 Type** | **Relative abundance (%)** |
| --- | --- | --- |
| **Bahrain (high latitude & thermal regime)** | | |
| *Cyphastrea microphtalma* | C15h-C15k-C15q-C15p | 0.72 |
|  | C3-C3bs-C3gulf-C3ef-C3c | 82.15 |
|  | C3-C3gulf-C3c-C3aj | 1.91 |
|  | C3/C3c-C3gulf | 15.21 |
| *Dipsastraea speciosa* | C3/C3c-C3gulf | 100.00 |
| *Platygra daedalea* | A1 | 3.63 |
|  | C3-C3u-C3gulf | 56.06 |
|  | C3/C3c-C3gulf | 7.38 |
|  | C3by | 1.09 |
|  | D1-D2.2-D1m | 0.20 |
|  | D5-D5a-D4-D5e-D4b-D4a | 31.63 |
| *Porites lutea* | C15 | 1.67 |
|  | C3-C3gulf-C3ar-C3as | 29.79 |
|  | C3-C3gulf-C3c-C3ed | 0.29 |
|  | C3/C3c-C3gulf | 38.58 |
|  | C3/C3gulf | 28.87 |
|  | C40f | 0.20 |
|  | C7 | 0.60 |
| *Tubinaria peltata* | C3-C3bs-C3gulf-C3ef-C3c | 100.00 |
| **Seychelles (mid latitude & moderate thermal regime)** | | |
| *Acropora gemmifera* | C15 | 0.03 |
|  | C1d/C1-C42.2-C1b-C3cg-C45c-C115k | 11.45 |
|  | C3z/C3-C115 | 5.60 |
|  | D1-D2.2-D1m | 82.9 |
| *Acropora muricata* | C3/C115/C3u-C115a-C115e-C21ab | 0.94 |
|  | C3z-C3-C3.10-C3an-C115-C3bq | 16.91 |
|  | C3z/C3-C115 | 1.45 |
|  | D1-D2.2-D1m | 43.62 |
|  | D1-D2.2-D4-D1m-D1c | 28.61 |
|  | D1-D4-D4c-D1c-D2 | 8.47 |
| *Dipsastraea speciosa* | C1-C1c-C1b-C1al-C42.2 | 1.75 |
|  | C1/C1c | 0.03 |
|  | C1/C1c-C1b | 0.18 |
|  | C15-C15dl | 1.75 |
|  | C3/C115/C3u-C115a-C115e-C21ab | 61.00 |
|  | **C3/C3gulf** | **0.12** |
|  | C3/C3u-C115 | 4.29 |
|  | D1-D2.2-D1m | 9.04 |
|  | D1-D4-D4c-D1c-D2 | 21.84 |
| *Favites pentagona* | C1b/C3-C1u | 100.00 |
| *Pavona cactus* | C1b/C3-C1u | 100.00 |
| *Porites lutea* | C15 | 3.78 |
|  | C15-C15ad-C15ai | 86.16 |
|  | C15-C15az-C15m | 0.18 |
|  | C15-C15dl | 0.26 |
|  | C15/C116 | 8.37 |
|  | C15/C15h/C116 | 0.19 |
|  | C3z | 0.33 |
|  | C3z/C3-C115 | 0.57 |
|  | D4r | 0.16 |
| **Indonesia (low latitude & thermal regime)** | | |
| *Acropora gemmifera* | C21-C21ag-C3-C21af | 11.29 |
|  | C40-C3-C115 | 88.71 |
| *Acropora muricata* | C15-C15l-C15n-C15bb-C15.8 | 2.74 |
|  | C21 | 9.15 |
|  | C21-C21ag-C3-C21af | 74.33 |
|  | C3 | 0.18 |
|  | C40-C3-C115 | 13.58 |
|  | D1/D4-D4c-D4f | 0.02 |
| *Dipsastraea speciosa* | C21 | 2.62 |
|  | C21-C21ag-C3-C21af | 10.81 |
|  | C3/C115/C3u-C115a-C115e-C21ab | 2.94 |
|  | C40-C3-C115 | 83.17 |
|  | D1-D4-D4c-D4f-D3b-D1c | 0.39 |
|  | D1/D4/D4c | 0.07 |
| *Favites pentagona* | C1/C42.2/C1b | 2.78 |
|  | C40-C3-C115 | 97.22 |
| *Pocilliopora damicornis* | C1d/C1-C42.2-C1b-C3cg-C45c-C115k | 92.01 |
|  | C40/C3 | 4.09 |
|  | D1/D2d | 3.90 |
| *Pocillopora verrucosa* | C15h | 0.87 |
|  | C1d/C1-C42.2-C1b-C3cg-C45c-C115k | 64.86 |
|  | C42.2/C1-C1b-C1au | 13.78 |
|  | D1/D2d | 20.49 |
| *Porites lutea* | C116a | 0.71 |
|  | C15-C15bn-C15by | 8.78 |
|  | C15-C15by-C15ai | 50.40 |
|  | C15-C15l-C15n-C15bb-C15.8 | 13.60 |
|  | C15h/C15 | 26.50 |

**Table S3.2.** Results of PERMANOVA on Principal Coordinate Analysis (PCoA) of Symbiodiniaceae ITS2 community composition across coral host species, thermal regimes, individual sites, and latitudinal transects. Analyses were conducted separately for *Cladocopium* (clade C) and *Durusdinium* (clade D). Significant *p-values* (*p<0.05*) are marked in (*), (*p<0.001*) are marked in (**) and (*p<0*) are marked in (***).

| **Model** | **PERMANOVA p-value** |
| --- | --- |
| *Cladocopium* (clade C) | |
| Between coral host species | F _10,116_ = 4127.8, R^2^ = 0.99, P = 9.999e-05 *** |
| Coral host species* thermal regime | F _10,116_ = 2261.17, R^2^ = 0.99, P = 0.001 ** |
| Coral host species* individual site | F _10,116_ = 4459.5, R^2^ = 0.99, P = 0.001 ** |
| Coral host species* latitude | F _10,116_ = 1209.3, R^2^ = 0.99, P = 0.04* |
| *Durusdinium* (clade D) | |
| Between coral host species | F _6,38_ = 478.2, R^2^ = 0.99, P = 3e-04 *** |
| Coral host species* thermal regime | F _6,38_ = 511.5, R^2^ = 0.99, P = 0.001 ** |
| Coral host species* individual site | F _6,38_ = 466.1, R^2^ = 0.99, P = 0.01 * |
| Coral host species* latitude | F _6,38_ = 1009.5, R^2^ = 0.99, P = 0.0002 *** |

**References:**

Davies, S. W., Ries, J. B., Marchetti, A., and Castillo, K. D. (2018). “Symbiodinium Functional Diversity in the Coral *Siderastrea siderea* Is Influenced by Thermal Stress and Reef Environment, but Not Ocean Acidification.” *Frontiers in Marine Science,* 5: 1–14.

Hansen, G., and Daugbjerg, N. (2009). “*Symbiodinium natans sp.* *nov*.: A “Free‐Living” Dinoflagellate From Tenerife (Northeast‐Atlantic Ocean).” *Journal of Phycology*, 45: 251–263.

Hume, B.C.C., D’Angelo, C., Smith, E.G., Stevens, J.R., Burt, J. & Wiedenmann, J. (2015) Symbiodinium thermophilum sp. nov., a thermotolerant symbiotic alga prevalent in corals of the world’s hottest sea, the Persian/Arabian Gulf. *Scientific Reports, 5*, 8562.

Jeong, H. J., Lee, S.Y., Kang, N. S. et al. (2014) Genetics and morphology characterize the dinoflagellate Symbiodinium voratum, n. sp., (dinophyceae) as the sole representative of Symbiodinium clade E. *Journal of Eukaryotic Microbiology, 61*, 75–94.

LaJeunesse, T.C., Parkinson, J.E., Reimer, J.D. (2012). A genetics-based description of Symbiodinium minutum sp. nov. and S. psygmophilum sp. nov. (Dinophyceae), two dinoflagellates symbiotic with cnidaria. *Journal of Phycology*. 48(6):1380-91. doi: 10.1111/j.1529-8817.2012.01217.x

Lajeunesse, T. C., Wham, D. C., Pettay, D. T., Parkinson, J. E., Keshavmurthy, S., & Chen, C. A. (2014). Ecologically differentiated stress-tolerant endosymbionts in the dinoflagellate genus Symbiodinium (Dinophyceae) Clade D are different species. *Phycologia*, *53*(4), 305-319. <https://doi.org/10.2216/13-186.1>

LaJeunesse, T. C., Lee, S. Y., Gil-Agudelo, D. L., Knowlton, N., & Jeong, H. J. (2015). *Symbiodinium necroappetens* sp. nov. (Dinophyceae): an opportunist ‘zooxanthella’ found in bleached and diseased tissues of Caribbean reef corals. *European Journal of Phycology*, *50*(2), 223–238. <https://doi.org/10.1080/09670262.2015.1025857>

LaJeunesse, T. C., Parkinson, J. E., Gabrielson, P. W., Jeong, H. J., Reimer, J. D., Voolstra, C. R., & Santos, S. R. (2018). Systematic Revision of Symbiodiniaceae Highlights the Antiquity and Diversity of Coral Endosymbionts. *Current biology, 28*(16), 2570–2580.e6. https://doi.org/10.1016/j.cub.2018.07.008

Lee, S.Y., Jeong, H.J., Kang, N.S., Jang, T.Y., Jang, S.H., Lajeunesse, T.C. (2015). *Symbiodinium tridacnidorum sp. nov*., a dinoflagellate common to Indo-Pacific giant clams, and a revised morphological description of *Symbiodinium microadriaticum* freudenthal, emended Trench & Blank. *European Journal of Phycology***, 50**: 155–172.

Parkinson, J.E., Coffroth, M.A., Lajeunesse, T.C. (2015). New species of Clade B Symbiodinium (Dinophyceae) from the greater Caribbean belong to different functional guilds: *S. aenigmaticum sp. nov*., *S. antillogorgium sp. nov*., *S. endomadracis sp. nov*., and *S. pseudominutum sp. nov*. *Journal of Phycology*, 51: 850–858.

Ramsby, B. D., Hill, M. S., Thornhill, D. J., Steenhuizen, S. F., Achlatis, M., Lewis, A. M., & LaJeunesse, T. C. (2017). Sibling species of mutualistic Symbiodinium clade G from bioeroding sponges in the western Pacific and western Atlantic oceans. *Journal of phycology*, *53*(5), 951–960. <https://doi.org/10.1111/jpy.12576>

Trench, R.K., Thinh, L. (1995). *Gymnodinium linucheae sp. nov*.: The dinoflagellate symbiont of the jellyfish *Linuche unguiculata*. *European Journal of Phycology*, 30: 149–154.

Trench, R. (2000). Validation of some currently used invalid names of dinoflagellates. *Journal of Phycology*, 36: 972.

Wham, D.C., Ning, G., LaJeunesse, T.C. (2017). *Symbiodinium glynnii sp. nov* ., a species of stress-tolerant symbiotic dinoflagellates from pocilloporid and montiporid corals in the Pacific Ocean. *Phycologia*, 56: 396–409.
